# Intelligent Active Suspension Control Method Based on Hierarchical Multi-Sensor Perception Fusion

**DOI:** 10.3390/s25154723

**Published:** 2025-07-31

**Authors:** Chen Huang, Yang Liu, Xiaoqiang Sun, Yiqi Wang

**Affiliations:** Institute of Automotive Engineering, Jiangsu University, Zhenjiang 212013, China; huangchen@ujs.edu.cn (C.H.); sxq@ujs.edu.cn (X.S.); 2212338019@stmail.ujs.edu.cn (Y.W.)

**Keywords:** multi-sensor, intelligent suspension, mode-switching, BP-PID control

## Abstract

Sensor fusion in intelligent suspension systems constitutes a fundamental technology for optimizing vehicle dynamic stability, ride comfort, and occupant safety. By integrating data from multiple sensor modalities, this study proposes a hierarchical multi-sensor fusion framework for active suspension control, aiming to enhance control precision. Initially, a binocular vision system is employed for target detection, enabling the identification of lane curvature initiation points and speed bumps, with real-time distance measurements. Subsequently, the integration of Global Positioning System (GPS) and inertial measurement unit (IMU) data facilitates the extraction of road elevation profiles ahead of the vehicle. A BP-PID control strategy is implemented to formulate mode-switching rules for the active suspension under three distinct road conditions: flat road, curved road, and obstacle road. Additionally, an ant colony optimization algorithm is utilized to fine-tune four suspension parameters. Utilizing the hardware-in-the-loop (HIL) simulation platform, the observed reductions in vertical, pitch, and roll accelerations were 5.37%, 9.63%, and 11.58%, respectively, thereby substantiating the efficacy and robustness of this approach.

## 1. Introduction

An intelligent suspension system constitutes an active suspension framework that leverages integrated operation among distributed sensors, control units, and actuators to continuously modulate suspension stiffness and damping coefficients in real time. The system architecture incorporates heterogeneous sensing modalities, including IMU, GPS, LiDAR, Camera, Radar and so on. Sensor fusion algorithms—such as Kalman filters and deep neural networks—are employed to attenuate sensor noise and correct for measurement inaccuracies, thereby enhancing state estimation fidelity [1,2,3,4,5,6,7].

The principal aims of sensor fusion in active suspension control systems are as follows: (1) Elimination of single-sensor limitations: For instance, gyroscopes exhibit drift errors requiring complementary accelerometer data; cameras become ineffective in low-light conditions, necessitating radar augmentation. (2) Enhancement of data reliability: Through redundant architectures (such as cross-validation between multiple accelerometers) to mitigate failure risks. (3) Implementation of predictive control: Combining GPS and map data to anticipate road conditions and proactively adjust suspension parameters [8,9,10,11,12].

The prevailing hierarchical levels and methodologies for multi-sensor fusion are as follows: (1) Data-level fusion entails the direct amalgamation of unprocessed sensor outputs—such as employing Kalman filtering to integrate accelerometer and gyroscope data—maximizing information preservation at the expense of increased computational demands. (2) Feature-level fusion, which combines extracted key features including vehicle chassis vibration spectra and road surface roughness metrics, prior to integration, thereby achieving an optimal trade-off between computational efficiency and fusion accuracy. (3) Decision-level fusion independently processes each sensor’s output and synthesizes the results via a centralized decision-making module, such as leveraging Bayesian inference frameworks to ascertain the necessity for active suspension system engagement [13,14,15,16,17,18].

A significant number of scholars have conducted extensive research on the application of visual cameras in vehicles. Schreiber D et al. [19] proposed a lane detection and tracking approach using a single camera, capable of switching between linear and nonlinear methods. The method leverages vehicle motion information, specifically yaw angle, to enhance detection robustness and accuracy. Kim J G et al. [20] proposed a lane detection method for autonomous vehicles based on stereo cameras. The approach utilizes disparity maps to effectively segment the road from surrounding vehicles, enabling lane detection within the isolated road regions. Experimental results indicate that the method maintains accurate road segmentation even in the presence of vehicles, and lane detection within these segmented areas is successfully achieved through the application of dynamic programming and Kalman filtering. Špoljar D et al. [21] proposed a novel algorithm for lane detection (LD) and lane departure warning (LDW) that operates exclusively on images captured by a front-mounted camera. The algorithm detects the number and spatial positions of lane markings within the image, while also identifying the current driving lane and the two adjacent lanes. In the event of lane departure, the system provides corresponding warning messages to the driver.

Lam et al. [22] proposed an algorithm capable of extracting urban surface elevation information from data acquired by a moving LiDAR sensor. The method involves segmenting the road surface into multiple planar regions, followed by the application of a Kalman filter to determine the centroid and orientation of each plane. Subsequently, a RANSAC (Random Sample Consensus) algorithm is employed to fit mathematical models to the planar segments. Experimental results demonstrate the effectiveness and robustness of the proposed approach. Jeremy J. Dawkins et al. [23] developed a terrain scanning system integrating LiDAR, GPS, and inertial navigation sensors. The experimentally measured road surface elevations were used as input for vehicle suspension system simulations. Comparative analysis between the simulated and actual road surfaces demonstrated that the simulated terrain could effectively substitute the real surface as input for suspension modeling, validating the feasibility and accuracy of the proposed approach. Vu et al. [24] employed LiDAR data for real-time recognition and classification of traffic signs. The system integrated onboard sensors, including a GPS/IMU platform, 3D LiDAR, and visual sensors. Virtual scan images were utilized to perform intensity filtering of the data points, followed by an analysis of the spatial relationships between high-intensity planar regions. Only those planes with inter-plane distances exceeding 1 m were retained for further processing. Seok J. [25] proposed a laser-based method for estimating road surface height profile splines and a pavement bump detection technique tailored for preview-based suspension systems. This approach enables accurate estimation of road elevation contours and detection of surface irregularities even under conditions of sparse LiDAR data. Through the above research, it has been identified that current multi-source sensor data fusion faces several challenges, including sensor data inconsistency issues, robustness in extreme environments, and concerns related to cost and power consumption.

To tackle the identified challenges, this study introduces an advanced active suspension control methodology leveraging multi-sensor hierarchical perception and data fusion techniques. The main contributions of this paper are summarized as follows:

(1) A binocular vision system is deployed for object recognition, enabling the detection of lane curve origins and speed bumps, as well as the acquisition of real-time distance measurements.
(2) An integrated GPS/IMU navigation module and LiDAR are employed to extract forward-looking road elevation profiles.
(3) A BP-PID control architecture is then developed to enable adaptive mode transitions of the active suspension system across three distinct roadway scenarios: flat surfaces, curved trajectories, and obstacle road. Furthermore, an ant colony optimization algorithm is implemented to optimize four key suspension parameters. The proposed approach is rigorously evaluated through simulation environments.
(4) A hardware-in-the-loop (HIL) simulation testbed incorporating the Development to Prototype (D2P) controller was established to validate the controller’s efficacy and operational robustness.

## 2. Target and Distance Recognition by Binocular Camera

### 2.1. Lane Line Detection Method Based on Image Processing

The prerequisite for identifying the starting position of the lane curve using a binocular camera is the successful detection of lane markings in the image. This study employs a traditional image processing pipeline, which includes preprocessing, lane detection, and lane fitting [26].

(1) Preprocessing

**Figure 1:**
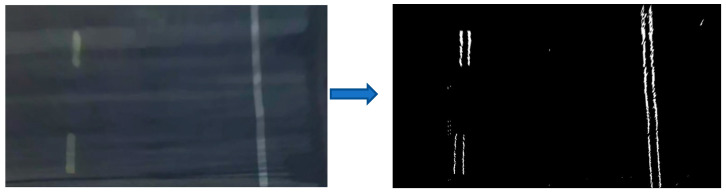
Aerial view of driveway line preprocessing.

Preprocessing plays a crucial role in lane detection, aimed at improving image quality and enhancing salient features. The preprocessing pipeline for lane lines encompasses raw image enhancement, region of interest (ROI) extraction, inverse perspective mapping, and binary edge detection, ultimately yielding an aerial-view lane line representation as illustrated in Figure 1.

(2) Lane line positioning

**Figure 2:**
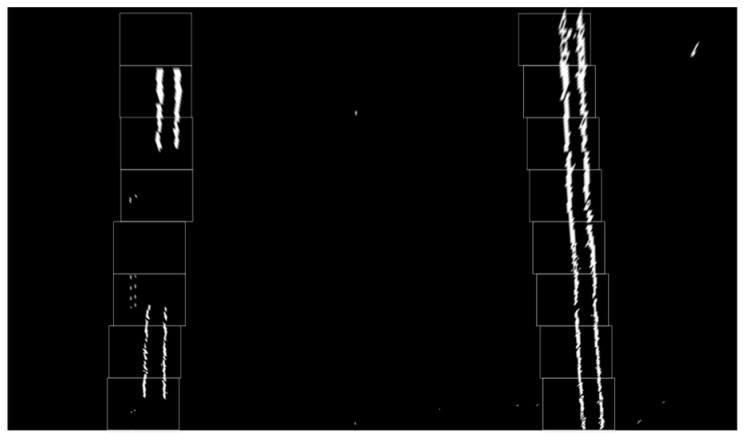
Lane line positioning effect.

After preprocessing, the aerial-view image displays white boundary lines on a black background. Lane identification is performed by detecting the coordinates of the white pixels. In this investigation, the sliding window technique [27] is utilized for lane extraction. The outcomes are presented in Figure 2.

(3) Lane line fitting

**Figure 3:**
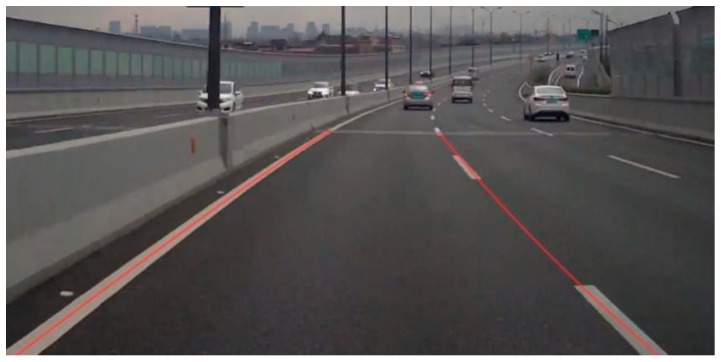
Lane line fitting effect.

The objective of lane fitting is to convert the identified discrete lane pixels into a continuous mathematical curve, thereby providing a precise representation of the lane’s position, geometry, and curvature. In this investigation, the least squares algorithm is utilized for lane fitting within the aerial view. The fitted lane lines generated through this method are illustrated in Figure 3.

**Figure 4:**
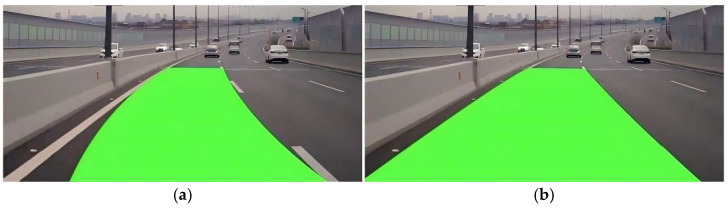
Comparison of Kalman filtering effects. (**a**) No Kalman filter. (**b**) Kalman filter.

The Kalman filter algorithm represents a discrete-time optimal state estimation methodology that synthesizes historical datasets with current observations to mitigate errors inherent in least squares approximation, thereby yielding enhanced precision in lane line modeling [28]. Figure 4 illustrates a comparative analysis of the outcomes, evidencing that Kalman filter implementation significantly augments the robustness of lane detection systems.

### 2.2. Lane Line Round-Curve Start-Point Position Detection

In this study, the initial point of a circular curve is identified using the lane markings of a well-defined branch road within an urban roadway network. Reference is made to the “Specifications for Highway Geometric Design” [29] to assess whether the radius of curvature exceeds 1000 m, which serves as the criterion for classifying a curve as circular. The calculation of the radius of curvature is performed using the following formula:

(1) $$k=\frac{y^{\prime \prime }}{{\left[1+{\left(y^{\prime }\right)}^{2}\right]}^{\frac{3}{2}}}$$

Let the location of the start point of the circular curve under the image coordinate system be ($x_{s}$, $y_{s}$), and the expression of the fitted curve of the lane line obtained according to the Kalman filter is as follows:

(2) $$y=a_{f}x^{2}+b_{f}x+c_{f}$$

where ($a_{f}$,$\;b_{f}$,$\;c_{f}$) represent the state variables.

Combining Equation (1) and differentiating gives the following:

(3) $$\frac{2a_{f}}{{\left[1+{\left(2a_{f}x_{s}+b_{f}\right)}^{2}\right]}^{\frac{3}{2}}}<\frac{1}{1000}$$

**Figure 5:**
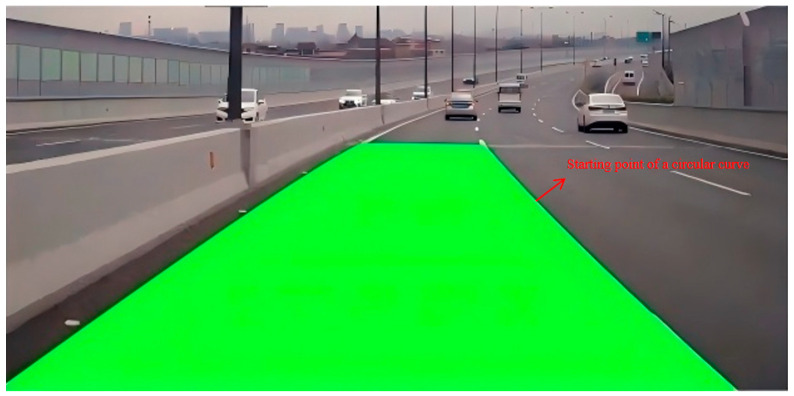
Circular-curve start-point recognition results.

Obtaining the range from the above formula and then selecting the maximum value, the positioning of the fitting curve can be obtained as ($x_{s}$, $y_{s}$). The completed effect is shown in Figure 5.

### 2.3. Speed Bump Position and Distance Detection

#### 2.3.1. Speed Bump Detection Model

**Figure 6:**
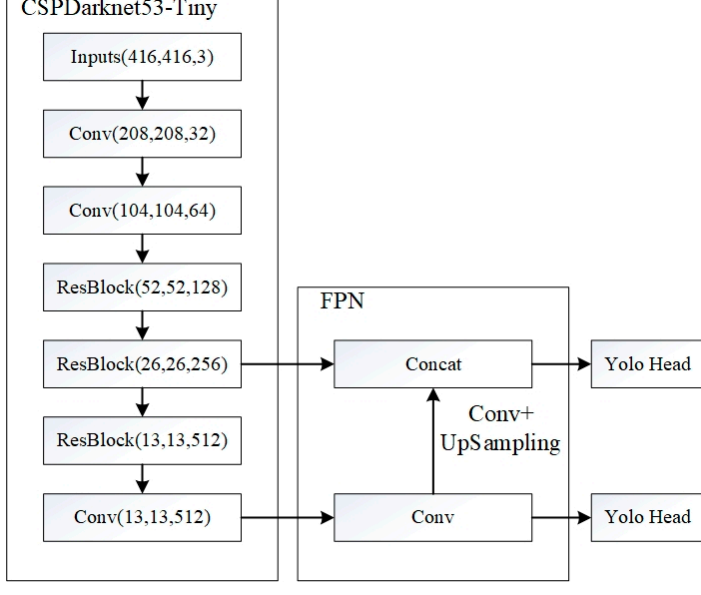
YOLOv4-tiny framework.

**Figure 7:**
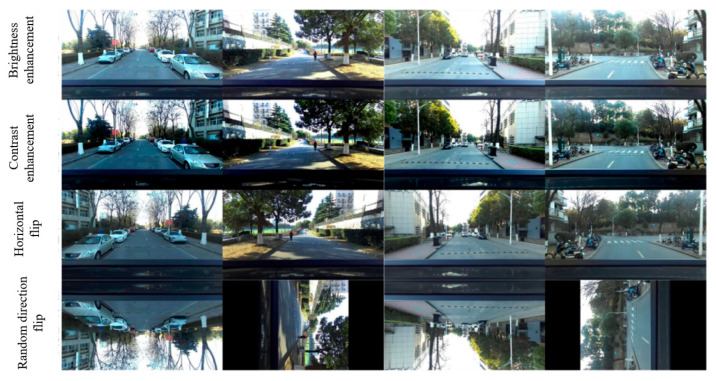
Enhanced datasets.

This study employs lightweight YOLOv4-tiny architecture, an optimized derivative of YOLOv4 designed to streamline feature extraction and improve computational efficiency, thereby fulfilling the real-time detection demands for speed bumps during vehicular operation, for which the framework diagram is shown in Figure 6. Due to the absence of publicly available speed bump datasets, real-vehicle image acquisition was conducted for this study. The collected SVO-format video streams were converted into MP4 video streams, from which the left and right images of each frame were extracted and saved. Speed bump images captured from various angles and under different exposure conditions were selected, and after manual screening, 1882 valid data samples were finally obtained. Through data augmentation techniques such as brightness and contrast enhancement, the dataset was further enriched, expanding it to a total of 4810 samples [30], as depicted in Figure 7. The dataset was then annotated using the LabelImg tool to produce VOC-compliant labels. Finally, the data was stratified into training, validation, and test subsets in a 7:2:1 ratio, thereby finalizing the dataset preparation protocol.

#### 2.3.2. Speed Bump Distance Detection

**Figure 8:**
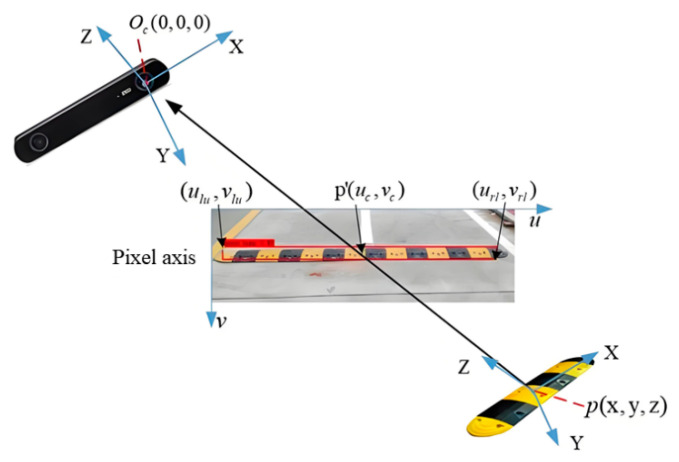
Reduction belt distance detection.

This study employs the centroid of the object detection bounding box as the reference for range estimation. Upon identification of a speed bump by the stereo vision system, the bounding box’s upper-left and lower-right coordinates are represented as ($u_{lu}$,$\;v_{lu}$) and ($u_{rl}$,$\;v_{rl}$), respectively. The centroid coordinates of the speed bump, ($u_{c}$,$\;v_{c}$), are computed from these vertices according to Equations (4) and (5). The corresponding schematic is illustrated in Figure 8.

(4) $$u_{c}=\frac{\left(u_{rl}-u_{lu}\right)}{2}+u_{lu}$$

(5) $$v_{c}=\frac{\left(v_{rl}-v_{lu}\right)}{2}+v_{lu}$$

**Figure 9:**
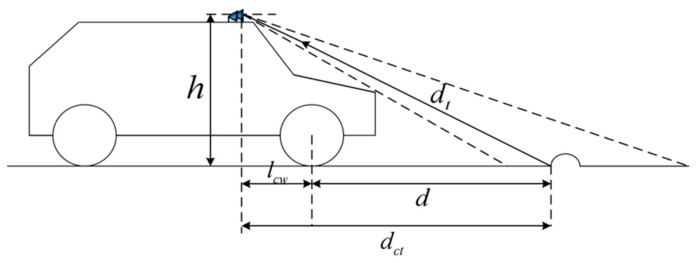
Target distance calculation method.

When a vehicle approaches a speed bump or a curve, detection of the speed bump position ($u_{c}$, $v_{c}$) or the starting point of the circular curve ($x_{s}$,$y_{s}$) allows determination of the actual distance between the target object and the vehicle through coordinate transformation relationships. Utilizing the depth perception capabilities of the ZED stereo camera, corresponding 3D point coordinates can be identified for target objects within the point cloud data. Therefore, knowing the pixel coordinates ($u_{c}$,$\;v_{c}$) or ($x_{s}$,$\;y_{s}$) of the target enables calculation of the actual distance $d_{t}$ from the camera to the target object. Taking speed bumps as an example, the method for calculating the distance between the vehicle and the target object after detection is shown in Equation (6), with the principle illustrated in Figure 9. The distance calculation for the starting point of a circular curve follows the same methodology.

(6) $$d=\sqrt{{d_{t}}^{2}-h^{2}}-l_{cw}$$

where h is the height of the camera from the horizontal road surface, and $l_{cw}$ is the horizontal distance from the camera to the center of the front axle.

## 3. Multi-Sensor Based on Elevation Pavement Information Extraction

**Figure 10:**
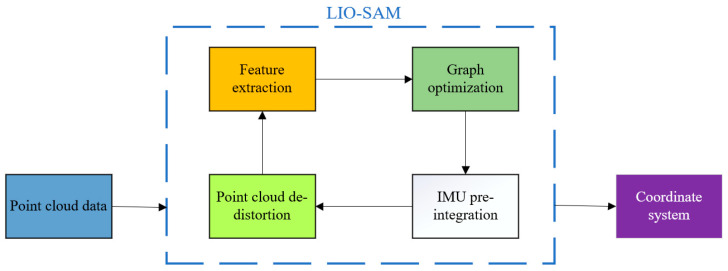
LIO-SAM algorithmic framework.

To generate a road surface model for the active suspension system, raw point cloud data is initially preprocessed using the Point Cloud Library (PCL). The LIO-SAM algorithm is then applied to the processed data to extract road elevation profiles along the vehicle’s trajectory [31]. This workflow incorporates modules such as point cloud de-distortion, feature extraction, graph optimization, and IMU pre-integration, as depicted in the system architecture in Figure 10. The LiDAR sensor deployed in this research is the RS-Ruby Lite 80-line LiDAR, while the integrated navigation platform comprises the X1-6H GNSS/INS/IMU high-precision tightly coupled navigation terminal.

**Figure 11:**
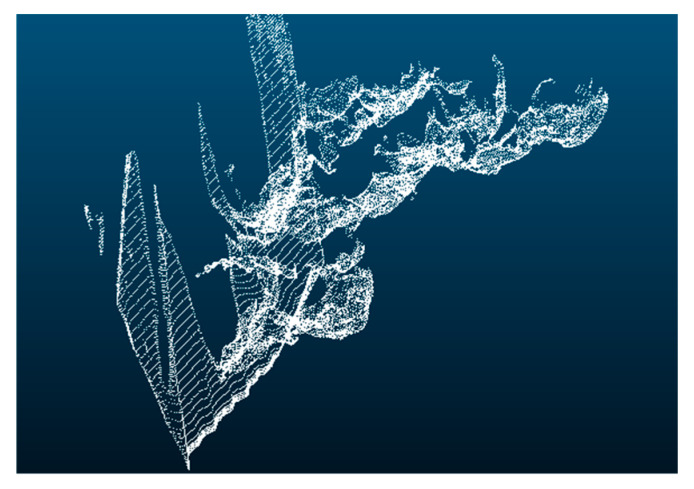
Vehicle front track position point cloud information.

The Passthrough filtering algorithm is a fundamental filtering method within the PCL, which retains points within a specified range along designated axes (e.g., x, y, z) while removing points outside the range and irrelevant data [32]. As illustrated in Figure 11, applying the Passthrough filter yields a point cloud representing the vehicle’s forward trajectory. Compared to the original unprocessed point cloud, this filtering step effectively eliminates extraneous elements like adjacent trees and buildings that do not pertain to road elevation, thereby functioning as an essential initial stage in point cloud data preprocessing.

**Figure 12:**
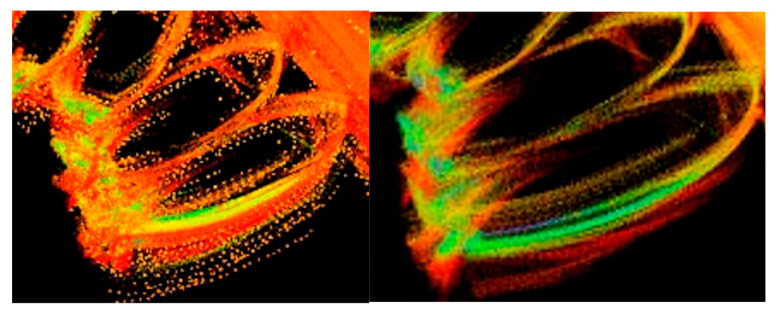
Point cloud de-distortion effect.

LIO-SAM enhances the LeGO-LOAM architecture by integrating tightly coupled IMU and GPS inputs. When preprocessed point cloud data is received from the PCL, the system initially executes distortion correction by fusing high-frequency IMU readings to enable motion compensation. This methodology mitigates intra-scan pose variations resulting from sensor dynamics and temporal misalignments during LiDAR data collection. The efficacy of this technique is demonstrated in Figure 12.

**Figure 13:**
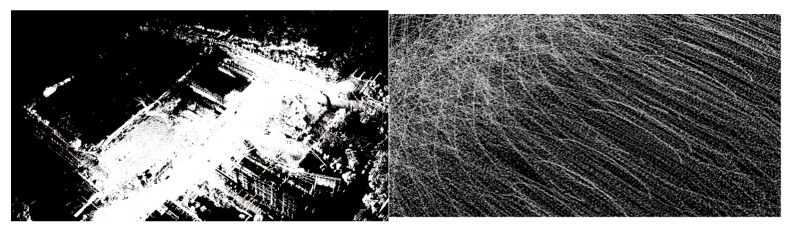
Three-dimensional point cloud effect.

Then, after feature extraction, graph optimization and IMU pre-integration, the final 3D point cloud obtained by the LIO-SAM algorithm is shown in Figure 13.

**Figure 14:**
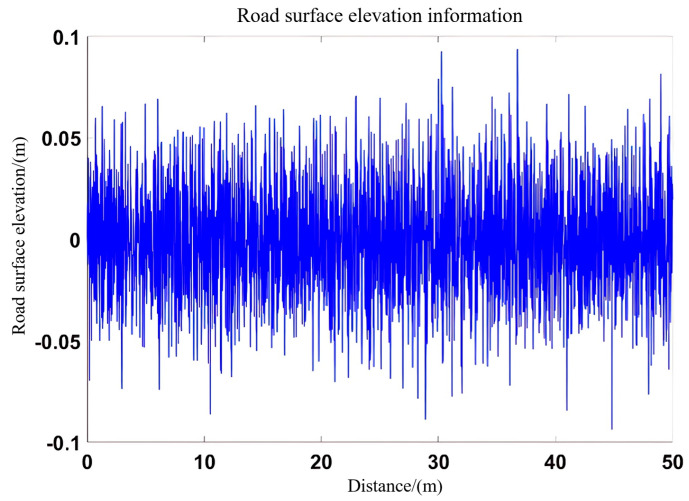
Elevation of the road surface at the location of the vehicle’s trajectory.

Finally, the ply file of the 3D point cloud is obtained, and the file is converted to mat format as a Simulink pavement model input. Figure 14 shows the converted pavement input model.

## 4. Multi-Sensor Based Intelligent Suspension Control Design

### 4.1. Multi-Sensor Based Intelligent Suspension Control Framework Construction

**Figure 15:**
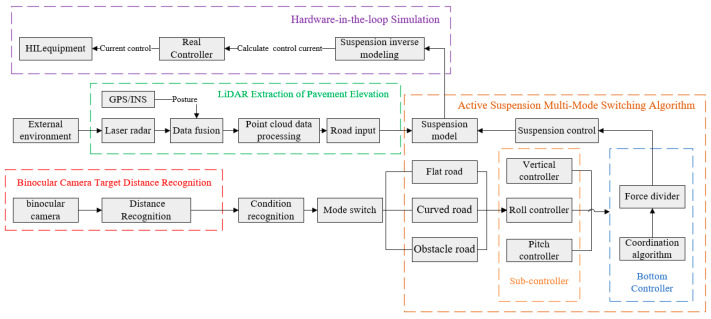
Control system framework.

The sensor pre-sensing architecture consists of a perception layer and an actuation layer. The perception layer integrates stereo cameras, LiDAR, inertial navigation system (INS), and GPS modules. The actuation layer comprises the suspension dynamics model and its associated controller. The stereo camera utilizes stereo vision algorithms to detect lane boundaries, calculates curvature-based distances to curve entry points, and leverages a YOLOv4 model to identify speed bumps, which inform suspension mode transitions. LiDAR acquires 3D point cloud data via multi-line scanning, and, together with INS attitude and GPS positioning data, transforms the point clouds into the global coordinate frame to generate a road surface elevation map. The GPS/INS integrated navigation system mitigates INS drift errors in real time, thereby improving the spatial accuracy of point cloud alignment. Outputs from the perception layer, such as road surface elevation, lane curvature, and obstacle detection, are transmitted to the actuation layer to control the active suspension system for adaptive road surface response. This system enables closed-loop control of vehicle dynamics and environmental adaptation through multi-sensor spatiotemporal calibration and data fusion. The proposed solution adopts a 7-DOF active suspension model, incorporating multi-sensor perception data, mode-switching logic, sub-controller modules, and a distribution framework, as depicted in Figure 15.

**Figure 16:**
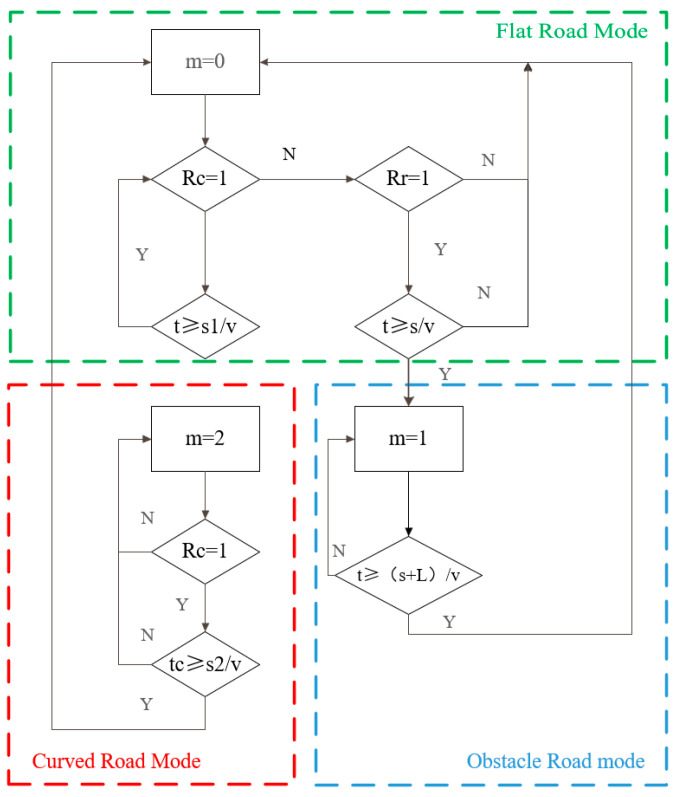
Pattern discrimination methods.

To ensure optimal ride comfort and handling performance under varying operating conditions, the suspension system in this study is categorized into three operational modes: flat road, curved road and obstacle road. The mode-switching logic diagram is shown in Figure 16.

Here, *m* is the working mode; *L* is the wheelbase; *v* is the vehicle speed; *Rr* is the result of speed bump recognition; *Rc* is the result of curve recognition; *s* is the distance; and *s*1 and *s*2 are the distances of the first and last recognition.

### 4.2. Online-Updated BP-PID Control

The back propagation (BP) neural network represents a class of multilayer feedforward neural architectures, optimized via the error back propagation algorithm. Its structural composition includes an input layer, multiple hidden layers, and an output layer, with each neuron interconnected through synaptic weights. During training, the network processes input data to produce an output, and the deviation between this output and the target value is utilized to iteratively update the synaptic weights and biases. This optimization cycle is repeated until the loss function converges to a predefined threshold.

The primary distinction between an online-updated BP neural network and a conventional BP neural network resides in their respective training methodologies. In traditional batch training, the model processes the entire dataset in a single epoch, after which the current weight parameters are stored, and subsequent training cycles commence using the same batch data. This method results in static weight parameters between training epochs. Conversely, an online-updated BP neural network employs incremental learning, wherein the model’s weights are dynamically adjusted with each incoming data instance. This enables the network to assimilate new information in real time, obviating the need for complete retraining and facilitating continuous adaptation to evolving data streams.

**Figure 17:**
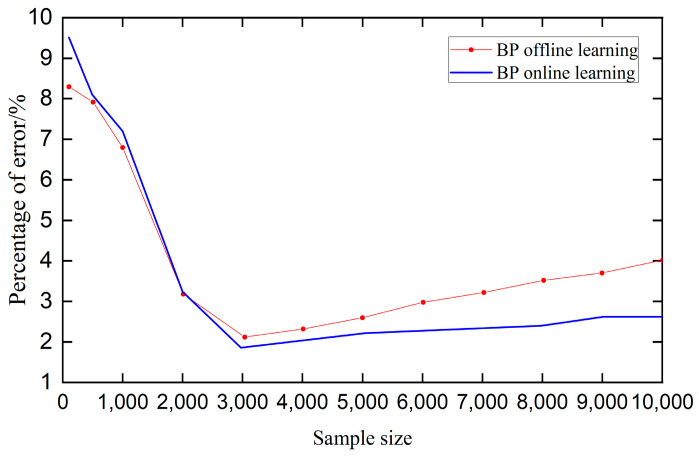
Comparison of error curves.

The neural network architecture is designed with input layers corresponding to the desired output *r*(*t*), the actual output *y*(*t*), and the error *e*(*t*). The hidden layer contains five neurons, while the output layer consists of three neurons representing $K_{p}$, $K_{i}$, and $K_{d}$, respectively. The sigmoid function is used as the activation function. To compare the traditional offline BP neural network with the online learning BP neural network, 10,000 sets of data were selected for comparative testing. Figure 17 presents a comparison of the error percentages between the offline and online learning neural networks.

As illustrated in Figure 17, when the sample size is below 2000, offline learning exhibits lower error rates compared to online learning. However, as the sample size increases, the error associated with online learning decreases markedly, eventually stabilizing at a level significantly lower than that of offline learning. Notably, once the sample size surpasses 3000, the error rate for online learning consistently falls below 0.03. Furthermore, demonstrates that online learning not only achieves a substantially faster convergence rate but also maintains lower error as the dataset grows. Consequently, for large-scale data, BP online learning emerges as the optimal approach.

### 4.3. Control System Co-Simulation

**Figure 18:**
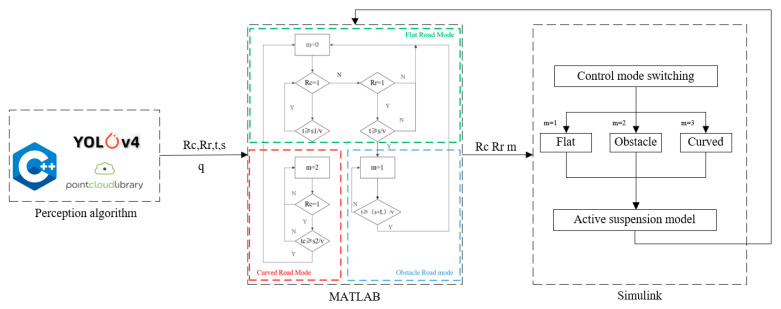
Co-simulation process.

Target distance estimation utilizing stereo vision and road elevation profiling via LiDAR are executed in C++, whereas the comprehensive vehicle suspension dynamics and control strategies are modeled in Simulink. The integrated co-simulation architecture is depicted in Figure 18. Outputs from the C++ modules—including the initial position of the circular trajectory (*Rc*), speed bump identification (*Rr*), timestamp (*t*), target range (*s*), and road surface irregularity (*q*)—are interfaced with MATLAB/Simulink 2021b. MATLAB processes these inputs to compute the mode transition parameter (*m*), facilitating adaptive control strategy selection. The finalized full vehicle suspension system and its corresponding control algorithms are implemented within Simulink.

### 4.4. Simulation Results Analysis

**Figure 19:**
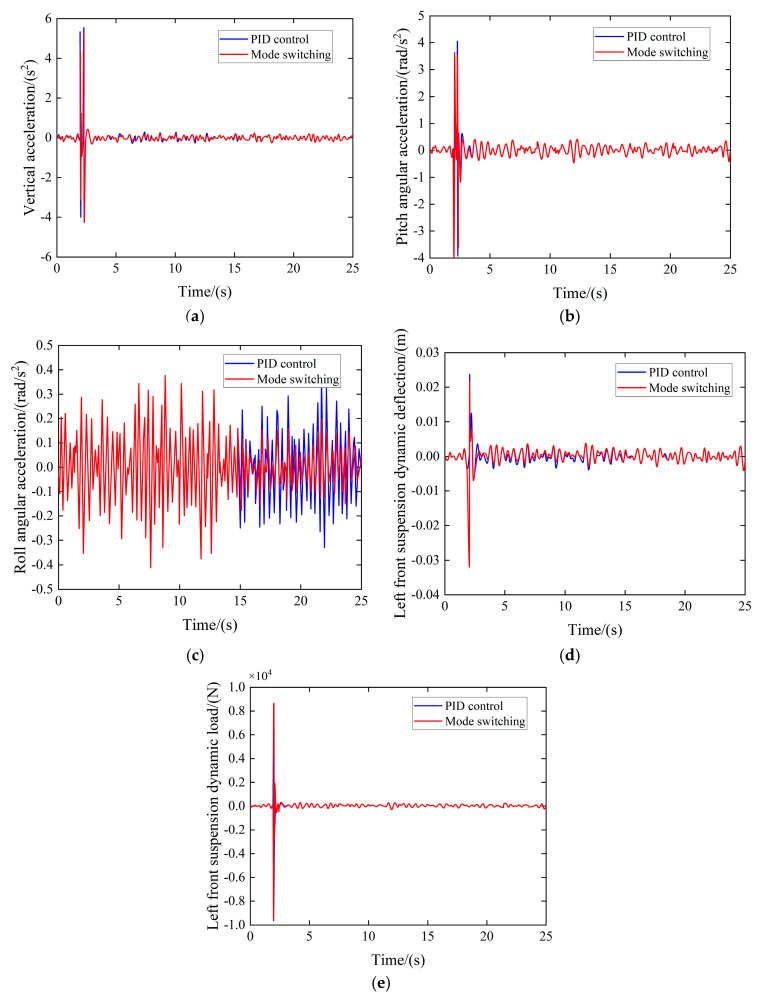
Control method co-simulation effect. (**a**) Vertical acceleration comparison. (**b**) Pitch angular acceleration comparison. (**c**) Roll angular acceleration comparison. (**d**) Left front suspension dynamic deflection comparison. (**e**) Left front suspension dynamic load comparison.

Based on the co-simulation results shown in Figure 19, the following can be seen:

During the time intervals of 0–2 s and 2.7–15 s, the vehicle was traveling along a straight road, while it traversed a speed bump within the 2–2.7 s interval and navigated a curve from 15 to 25 s. Compared to a traditional PID control strategy, the multi-mode switching control approach resulted in a reduction in the vehicle’s vertical acceleration RMS value by 7.43%, pitch acceleration RMS value by 12.09%, and roll angle acceleration RMS value by 14.47%. Additionally, throughout the entire simulation duration, the RMS value of the suspension’s dynamic deflection decreased from 0.0013 to 0.0012, representing a 7.39% reduction. The RMS value of the suspension’s dynamic load decreased from 86.93 to 81.35, corresponding to a 6.39% reduction. These results indicate a notable improvement in both metrics. A detailed comparison of the RMS values of various performance indicators is presented in Table 1.

## 5. Hardware-in-the-Loop Testing

**Figure 20:**
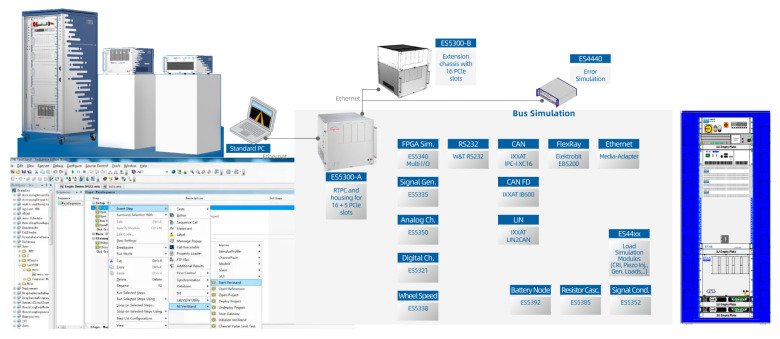
Diagram of HIL equipment at Vehinfo.

Hardware-in-the-loop (HIL) simulation is an advanced testing methodology that integrates physical hardware components with a virtual simulation environment. The hardware simulation platform comprises a Real-Time Processing Computer (RTPC), I/O boards, load modules, interconnection signal interfaces, and a fault injection unit. In this study, the HIL system provided by Vehinfo from Shanghai China was utilized, with LABCAR-OPERATOR (LCO) 3.8 employed as the host computer software. The constructed HIL testing environment consists of the LABCAR test cabinet, a host computer, and a D2P module. The LABCAR system is equipped with IO signal boards and CAN communication boards, enabling the transmission of required signals to both the controller and the host computer, as illustrated in Figure 20.

(1) Random pavement simulation

**Figure 21:**
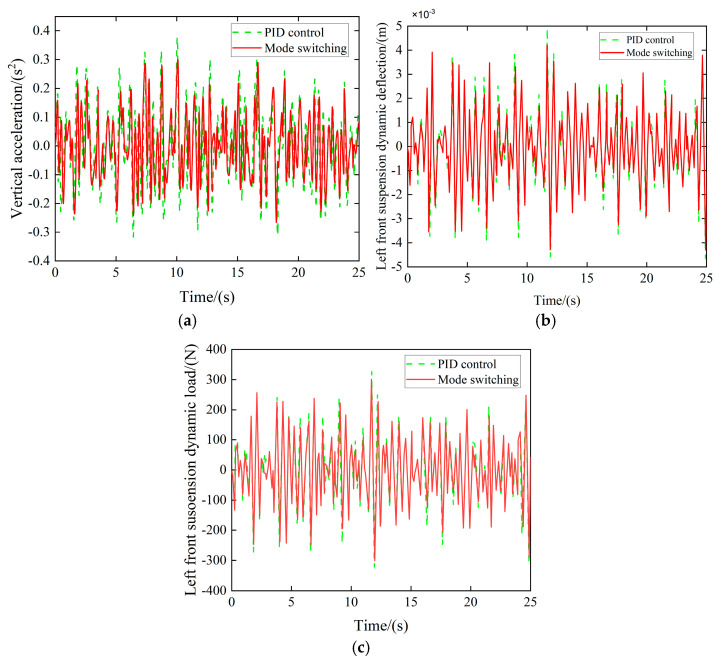
Vertical controller simulation effect. (**a**) Vertical acceleration comparison. (**b**) Left front suspension dynamic deflection comparison. (**c**) Left front suspension dynamic load comparison.

When the vehicle is traveling at a speed of 36 km/h on a Class B road surface, the control mode is switched to the flat road mode. The suspension vertical controller suppresses the vehicle body’s vertical motion, and the resulting performance metrics are presented in Figure 21.

Experimental results demonstrate that the multi-mode switching control strategy significantly enhances suspension vertical acceleration on flat road surfaces. The dynamic deflection and dynamic load of the left front suspension were reduced by 5.37%, 4.24%, and 4.12%, respectively, thereby validating the effectiveness of the controller.

(2) Obstacle road simulation

**Figure 22:**
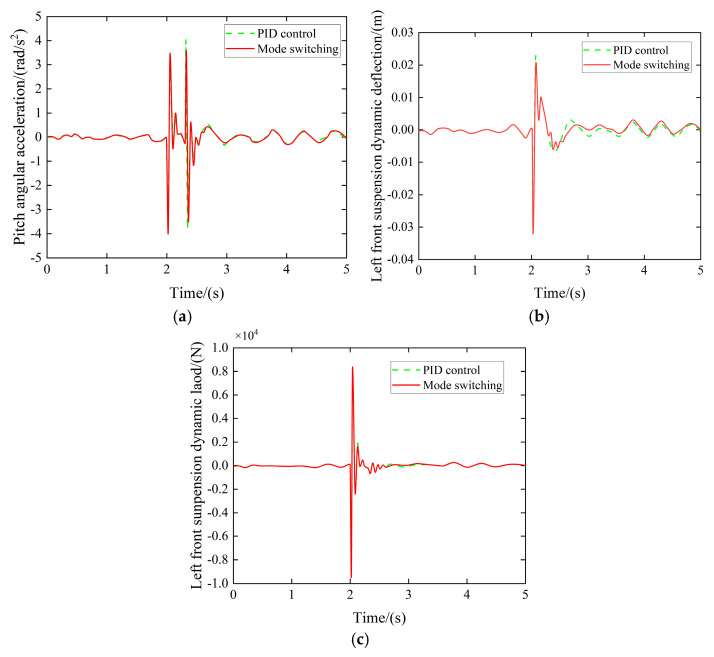
Pitch controller simulation effect. (**a**) Pitch angular acceleration comparison. (**b**) Left front suspension dynamic deflection comparison. (**c**) Left front suspension dynamic load comparison.

When the vehicle traverses the speed bump at 18 km/h, the control mode transitions to the obstacle road surface mode, and the suspension pitch controller suppresses vehicle body pitch motion. The resulting performance metrics are presented in Figure 22.

Experimental results indicate that when the vehicle traverses a speed bump, the peak value of the pitch angle during the acceleration phase is effectively reduced. While maintaining ride comfort, the dynamic deflection and dynamic load of the left front suspension are reduced by 9.63%, 4.52%, and 5.06%, respectively, thereby demonstrating the effectiveness of the multi-mode switching controller.

(3) Curved road simulation

**Figure 23:**
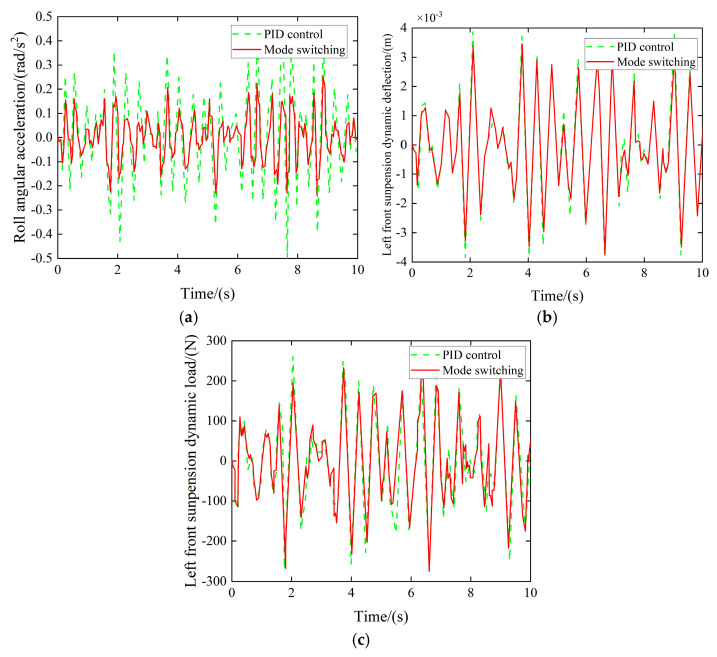
Roll controller simulation effect. (**a**) Roll angular acceleration comparison. (**b**) Left front suspension dynamic deflection comparison. (**c**) Left front suspension dynamic load comparison.

When the vehicle traverses the curve at 55 km/h, the controller transitions to the curved road surface mode, targeting the suppression of body roll. The resulting performance metrics are presented in Figure 23.

Experimental findings demonstrate that the proposed control algorithm significantly mitigated suspension performance metrics during lateral vehicle dynamics, with the left front quarter-car assembly exhibiting reductions of 11.58%, 3.73%, and 4.11% in dynamic deflection and dynamic load parameters, respectively. These quantitative improvements substantiate the efficacy of the implemented control strategy under cornering maneuvers.

Following experimental evaluation across three different road surfaces, the results demonstrate that the active suspension controller utilizing a multi-mode switching strategy substantially enhances vertical acceleration attenuation. Key performance indicators in both obstacle negotiation and cornering scenarios exhibit significant improvements, thereby substantiating the efficacy of the proposed controller. This methodology ultimately guarantees superior ride comfort and vehicle handling stability.

## 6. Conclusions and Prospects

This study addresses the limitations of existing active suspension research, specifically the inadequate integration of dynamic road condition variability into control performance indices, by introducing an intelligent suspension control framework leveraging multi-sensor data fusion. Real-time acquisition of road profile characteristics and vehicle state vectors is achieved through heterogeneous sensing modalities, including LiDAR and stereo vision systems, to construct a predictive information repository. A mode-switching adaptive control algorithm is subsequently developed, which incorporates previewed environmental parameters into the vehicle dynamics model. Comprehensive co-simulation validation reveals that, relative to traditional PID control, the proposed strategy achieves a 5.37% reduction in vertical acceleration, a 9.63% decrease in pitch acceleration, and an 11.58% reduction in roll acceleration. Furthermore, suspension dynamic deflection is reduced by over 4% across diverse road profiles, thereby improving ride comfort, handling stability, and overall vehicle dynamics. The closed-loop integration of previewed data and adaptive control parameters establishes an environment-adaptive architecture, enabling intelligent suspension systems to robustly manage complex and variable road conditions.

While the efficacy of the proposed methodology has been substantiated via simulation and HIL experiments, several areas warrant further optimization:

(1) Although both stereo vision systems and LiDAR sensors were employed in this investigation, a holistic sensor fusion of their respective datasets was not implemented. Given that stereo cameras can also reconstruct 3D point clouds, fusing point cloud data from both modalities could markedly improve the precision of road elevation mapping.
(2) Owing to certain limitations, the validation of the proposed control algorithm was confined to simulation and HIL testing. Comprehensive validation through full-scale vehicle trials is essential to further corroborate these results.

## Figures and Tables

**Table 1:** Comparison of RMS values of performance indicators.

RMS Values	PID Control	Mode Switching	Decrease
Vertical acceleration	0.0995	0.0921	7.43%
Pitch acceleration	0.1637	0.1439	12.09%
Roll angular acceleration	0.1396	0.1194	14.47%
Suspension dynamic deflection	0.0013	0.0012	7.69%
Suspension dynamic load	86.90	81.35	6.39%

## Data Availability

All data are contained within the article.

## Abbreviations

The following abbreviations are used in this manuscript:

GPS	Global Positioning System
IMU	Inertial Measurement Unit
LiDAR	Light Detection and Ranging
BP-PID	Back Propagation-Proportional Integral Derivative
HIL	Hardware-In-the-Loop
D2P	Development to Prototype
ROI	Region of Interest
PCL	Point Cloud Library
INS	Inertial Navigation System
LIO-SAM	Tightly coupled Lidar Inertial Odometry via Smoothing and Mapping
LeGO-LOAM	Lightweight and Ground-Optimized Lidar Odometry and Mapping
RMS	Root Mean Square
RTPC	Real-Time Processing Computer
CAN	Controller Area Network
LCO	LABCAR-OPERATOR
